# African Americans’ lived experiences with serious mental illness and cardiovascular disease: a syndemic perspective

**DOI:** 10.3389/fpsyt.2025.1711836

**Published:** 2025-12-10

**Authors:** Margaret Salisu, Carla Boutin-Foster, Laura Geer, Michele Pato

**Affiliations:** 1 College of Medicine, Downstate Health Sciences University, Brooklyn, NY, United States; 2 State University of New York (SUNY) Downstate Health Sciences University School of Public Health, New York, NY, United States; 3 Center for Psychiatric Health and Genomics (CPHG) Rutgers University, New Brunswick, NJ, United States

**Keywords:** syndemic theory, serious mental illness (SMI), structural drivers, social determinants of health, medical mistrust, African Americans (AA), health disparities, grounded theory

## Abstract

**Background:**

African Americans with serious mental illness (SMI) face a disproportionate burden of cardiovascular disease (CVD), reflecting the intersection of biological vulnerability and structural inequities. Yet little is known about how individuals living with both conditions understand and navigate these interconnections in their daily lives.

**Methods:**

Guided by grounded theory and syndemic theory, we conducted semi-structured interviews with 23 African American adults diagnosed with SMI and self-reporting CVD. Participants were recruited from the Genomic Psychiatry Cohort (GPC) and its African American sub-cohort (AAGPC). Interviews explored perceptions of illness interaction, self-management, and contextual barriers. Data were analyzed using constant comparative methods to identify patterns and develop a theoretical model of syndemic interdependency.

**Results:**

Analysis generated the core concept of syndemic interdependency—a dynamic process through which psychiatric and cardiovascular conditions interact and mutually reinforce one another within contexts of structural deprivation, Environmental and neighborhood influences, and Healthcare distrust. Participants described how psychiatric symptoms disrupted adherence to CVD management, while cardiovascular complications heightened psychiatric distress, creating self-perpetuating cycles of illness. Structural constraints such as reliance on public assistance and food insecurity provided minimal stability but reinforced dependency. Environmental influences, including unsafe housing and neighborhood violence, further undermined health behaviors. Distrust in healthcare providers—particularly in psychiatric care—emerged as a cross-cutting mechanism that deepened disengagement and exacerbated comorbid illness trajectories.

**Conclusion:**

This study extends syndemic theory by illustrating how individuals themselves conceptualize the reciprocal entanglement of SMI and CVD within structural and environmental constraints. Findings underscore the need for integrated, equity-focused care models that address psychiatric and cardiovascular health simultaneously while embedding trust-building, housing stability, and food security interventions. Disrupting SMI–CVD interdependency ultimately requires addressing the structural and institutional forces that sustain these overlapping health disparities.

## Introduction

African Americans in the United States face a disproportionate burden of serious mental illness (SMI) and cardiovascular disease (CVD) and related risk factors (1, 2), two critical and often co-occurring health conditions that have been linked to complex social, cultural, and psychological stressors (3, 4). SMI, such as schizophrenia, bipolar disorder, and schizoaffective disorder (5), and Cardiovascular disease (CVD) and related risk factors are associated with high morbidity and mortality rates and rank among the most socially and economically burdensome diseases in the United States (6, 7) Individuals diagnosed with SMIs (e.g., schizophrenia [SZ] or bipolar disorder [BD]) and with diabetes, high blood pressure, cholesterol, obesity, and other conditions potentially paired with habits such as smoking and/or excess drinking carry an increased risk of CVD (8).

CVD remains the leading cause of death among African Americans, with prevalence rates that surpass those seen in other ethnic groups (9–11). The co-occurrence of SMI with CVD and related conditions exacerbates individual health risks (12), complicates treatment adherence (13), and ultimately impairs quality of life (14), yet remains understudied (5).

Research indicates that social determinants, including racial discrimination, socioeconomic inequality, and inadequate access to healthcare, play an instrumental role in shaping health outcomes within this population (15, 16), which may impact their perceptions and responses to mental health and cardiovascular issues (17). Psychologically, stigma surrounding mental illness (18) create barriers to both diagnosis and effective treatment (19). The present study seeks to explore how African Americans with co-occurring SMI and CVD define, understand, and navigate their conditions within the context of these intersecting social, cultural, and psychological factors.

In this study, we apply the syndemic theory, which suggests that multiple, interrelated health issues are driven by and interact with social and environmental factors, to investigate the unique experiences of African Americans with both SMI and CVD (20). This approach allows for a comprehensive understanding of how overlapping conditions are not merely comorbid but synergistic, with each condition exacerbating the other, particularly in a context of social disadvantage. The insights gained from this research aim to inform culturally sensitive, holistic approaches to healthcare that address not only the clinical but also the social and cultural needs of African Americans facing dual diagnoses.

## Literature review

### Social cultural context of SMI and CVD

The prevalence of SMI and CVD among African Americans is shaped by social determinants that intersect with broader historical and systemic inequalities (21). Research has shown that as a result of structural racism, African Americans are more likely to experience socioeconomic hardships such as poverty, unemployment, and limited access to quality education, all of which have been linked to poor health outcomes (22, 23). These factors contribute to chronic stress, which increases vulnerability to both CVD and mental health disorders through pathways that involve the hypothalamic-pituitary-adrenal axis and inflammation (24).

Racial discrimination further compounds these health disparities by generating psychological stress, which has been associated with elevated blood pressure, inflammation, and higher rates of depressive symptoms (25). Chronic exposure to discrimination is known to have lasting effects on mental health, contributing to disorders such as anxiety and depression, which are risk factors for SMI. For example, in their study on discrimination and health, Williams et al. (26) found that African Americans who frequently encountered discrimination were more likely to report symptoms of psychological distress and physical illness, particularly CVD.

### Cultural and psychological factors influencing health perceptions

Cultural beliefs and community norms significantly influence how African Americans perceive and respond to both SMI and CVD. Snowden (27) highlights that African Americans may prioritize resilience and self-sufficiency, which are deeply embedded in cultural narratives of survival in the face of adversity. However, these values can also deter individuals from seeking mental health treatment due to concerns about stigma and the perceived need to “handle it on their own” (28). This hesitation is particularly pronounced in the African American community, where mental illness is often stigmatized and viewed as a weakness or failure (29). AA individuals with SMI who have CVD are marked by multiple stigmas (30) and are less likely to seek mental health treatments or services than White individuals.

The perception of mental illness as a personal, rather than medical, issue may lead to delays in diagnosis and treatment, with African Americans often only seeking mental health services once their conditions have become severe (31). Moreover, psychological stress from socioeconomic strain and exposure to violence can manifest in somatic symptoms, potentially leading individuals to seek medical treatment for physical ailments (like CVD) while ignoring or downplaying psychological symptoms (32). This dualistic view of health, where mental and physical health are often compartmentalized, complicates effective management of co-occurring conditions.

### The syndemic framework in health disparities research

Syndemic theory, first developed by Merrill Singer (20), is a conceptual framework that examines how co-occurring health conditions interact within a specific social and environmental context, often exacerbating each other’s effects. This theory has been applied to various health issues in marginalized populations, revealing how multiple diseases and social disadvantages intersect to produce unique health challenges that cannot be understood in isolation. For instance, studies using the syndemic model have highlighted how HIV, substance abuse, and violence among low-income populations create complex health challenges that are mutually reinforcing (33).

Within the context of African Americans with co-occurring SMI and CVD, syndemic theory provides a lens to explore how social stressors, cultural expectations, and mental health stigma contribute to a compounded health burden. Unlike traditional approaches to comorbidity, which treat each condition separately, the syndemic approach emphasizes the synergistic relationship between conditions like SMI and CVD, suggesting that each condition may worsen the course of the other, particularly in socially disadvantaged groups (34). By applying syndemic theory, we can better understand how African Americans experience and manage these dual diagnoses within a framework that considers the broader social determinants and cultural contexts that shape their health outcomes. The present study employs syndemic theory as a guiding framework to investigate how African Americans with SMI and CVD define and interpret their experiences, providing insights into how health systems can better respond to their unique needs.

This study explores the following research question: How do African Americans with co-occurring serious mental illness and cardiovascular disease define and understand their conditions within the context of their social, cultural, and psychological experiences? By employing syndemic theory and focusing on lived experiences, this research aims to uncover the multifaceted barriers and facilitators that shape the health outcomes of African Americans with dual diagnoses. Understanding these perspectives is critical for developing culturally sensitive, holistic healthcare approaches that acknowledge the intertwined nature of social and health inequalities within this population.

## Methods

### Participants and sampling

Participants were drawn from the Genomic Psychiatry Cohort (GPC) and its African American sub-cohort (AAGPC**),** large, ongoing studies of individuals with serious mental illness (SMI) designed to advance understanding of genetic, clinical, and environmental influences on psychiatric conditions. The design and procedures of both cohorts have been described extensively elsewhere (35, 36).

For this qualitative study, we employed a purposive sampling strategy to recruit a subset of participants who (1) self-identified as African American, and (2) reported a history of cardiovascular disease (CVD) or related cardiometabolic conditions such as hypertension or diabetes. Importantly, screening for SMI and CVD was conducted during the initial GPC enrollment process, which included standardized self-report questionnaires and structured diagnostic interviews by trained mental health professionals. No additional clinical screening was performed for this study. Instead, we recontacted eligible individuals from the GPC/AAGPC who had provided consent to be approached for future studies.

### Recruitment and participation

For this study, 125 eligible individuals who met study criteria were recontacted by telephone and informed about the purpose of the study. Of these, 45 were successfully reached, and 30 agreed to participate. Seven individuals were not ultimately interviewed—five withdrew citing loss of interest or scheduling difficulties, and two could not be recontacted due to disconnected numbers. The final sample included 23 participants, at which point theoretical saturation was determined; no new insights emerged, and subsequent interviews yielded repetition of core concepts. Interviews were audio-recorded, transcribed verbatim by the first author, and analyzed using grounded theory methodology to allow in-depth exploration of emergent categories and the development of a theoretical model.

### Sample characteristics and representativeness

Of the 23 participants, 14 identified as female and 9 as male, with an average age of 43.5 years. The sample was socioeconomically disadvantaged, with most relying on Supplemental Security Income (SSI) or food stamps. This demographic profile reflects the characteristics of the GPC’s urban service catchment area and aligns with the study’s purposive design—namely, to capture the lived experiences of individuals most affected by structural inequities, rather than to generalize to all African Americans with SMI and CVD. The age pattern observed was typical and had been reported in our earlier work on the GPC/CVD African American study (36), which documented the early onset and clustering of cardiometabolic disease among African Americans with SMI.

### Diagnostic verification and justification of measures

SMI diagnoses were confirmed via structured clinical interviews conducted by trained mental health professionals during initial GPC enrollment, including DSM-5–based diagnostic instruments for schizophrenia spectrum, bipolar, and major affective disorders. CVD status, by contrast, was self-reported during both the GPC baseline assessment and the present study. While this introduces some asymmetry in diagnostic rigor, the original GPC/AAGPC cohort from which these individuals were recontacted used a standardized medical screener where chronic conditions, including cardiovascular disease, were self-reported. This approach was consistent with prior GPC studies (35, 36). For the present study, participants were also asked during interviews to confirm their CVD status, indicate when they were first diagnosed, and describe how these conditions were being managed. The potential for reporting bias associated with self-report is acknowledged and addressed in the study’s limitation section.

### Grounded theory analysis

In this study, grounded theory analysis was applied to explore the nuanced, multi-dimensional health experiences of African Americans with co-occurring serious mental illness (SMI) and cardiovascular disease (CVD). Grounded theory, a qualitative research methodology developed by Mendenhall et al. (37), is particularly suited to studies like this one where the aim is to generate a theory grounded in the lived experiences and perspectives of participants. In this context, grounded theory provided a systematic framework for capturing the complex interplay between participants’ mental and physical health, socioeconomic status, stigma, healthcare distrust, and environmental challenges.

The grounded theory process in this study began with open coding, where the authors reviewed transcribed interview data line by line to identify initial codes that reflected significant phrases, concepts, and actions within participants’ narratives. Examples of codes from this stage included “commodification of medication”, “economic hardship” and “Impact of neighborhood on health”. Each code captured a piece of the participants’ perceptions and actions related to their health and healthcare engagement.

Following open coding, the authors engaged in axial coding to begin connecting these initial codes into broader categories or themes. For instance, codes related to healthcare distrust and skepticism toward providers were grouped under a category labeled “Distrust in the Healthcare System”. Similarly, codes like “inability to afford healthy food” and “reliance on subsidized housing” were connected under the theme of “Underlying Structural Drivers of Health Behaviors”. This stage of analysis enabled the authors to identify patterns within participants’ experiences, showing how various factors were interrelated and influenced their overall health management strategies.

The final stage, selective coding, involved integrating and refining these themes into a core theoretical concept: Syndemic Interdependency.

### Analytic process and coder roles

The analytic team included the first author (a qualitative researcher with clinical social work training and postdoctoral epidemiological training), and an advisor with expertise in public health and health equity, and two trained medical students with prior qualitative research experience who assisted with initial coding. The first author and the advisor jointly led the analytic process, while the medical students coded early transcripts to identify initial patterns and refine the preliminary codebook. The senior qualitative mentor provided oversight and served as adjudicator during theme consolidation.

### Consensus and inter-coder reliability

To strengthen analytic rigor, approximately 25% of the transcripts were double-coded by independent coders. Coding meetings were held weekly to review discrepancies, refine code definitions, and ensure consistency in interpretation. Divergent coding was discussed until theoretical consensus was reached, emphasizing conceptual coherence rather than numerical agreement. Peer-debriefing sessions with the senior mentor were conducted periodically to review emergent themes, test alternative interpretations, and verify that findings remained grounded in the data.

### Reflexivity

Reflexivity was integrated throughout the analytic process. Team members maintained reflexive memos documenting their positionality, assumptions, and emotional responses to participants’ narratives. The first author, an African American clinician–researcher, and the medical student coders, who represented diverse racial and gender backgrounds, engaged in ongoing reflection about how their positional and professional perspectives could shape data interpretation. These discussions were used to surface potential bias, challenge assumptions, and identify negative cases that did not align with dominant patterns.

### Saturation and validity checks

Theoretical saturation was monitored through three indicators: (1) stabilization of the codebook—no new codes emerging across successive transcripts; (2) redundancy in thematic properties—new interviews elaborated but did not alter core dimensions; and (3) use of saturation grids to track the recurrence of categories across participants. Saturation was considered achieved when new data confirmed rather than expanded existing categories, and when the central category—syndemic interdependency—coherently integrated all major themes.

Analytic rigor was further enhanced through maintenance of an audit trail (including meeting notes, memos, and codebook versions), peer debriefing, and transparent documentation of analytic decisions. This iterative, reflexive, and consensus-driven process ensured that findings were empirically grounded while maintaining methodological credibility and transparency.

Throughout, the analysis remained iterative and reflexive. Team members continuously revisited earlier transcripts and memos to refine categories and ensure theoretical saturation. This approach allowed for deep immersion in the data, ensuring that the final theory was directly rooted in participants’ actual words and experiences. Using software like Dedoose facilitated this analysis by enabling efficient organization and retrieval of codes and excerpts, making it easier to identify connections between different parts of the dataset.

By using grounded theory in this study, the authors were able to move beyond merely describing individual health challenges and instead develop a substantive theory that illustrates how these challenges interlock within a broader social context. This approach revealed a multi-layered understanding of health management in this population, highlighting the necessity for interventions that address not only medical but also social and environmental dimensions.

## Results

### Participant characteristics

Participant characteristics are summarized in **Table 1**. The sample (N = 23) ranged in age from 29 to 62 years, with a mean age of 43.5 years (SD = 8.8). This age distribution reflects a pattern typical of African American adults with co-occurring SMI and CVD, as previously reported in related work from the GPC/AAGPC cohort (36).

**Table 1:** Demographic and social determinants of health characteristics of participants (N = 23).

Characteristic	n (%) or mean (SD)
Age (years)	43.5 (8.8)
Age range	29–62
Sex	
Female	14 (60.9)
Male	9 (39.1)
Marital status	
Single/never married	13 (56.5)
Married/partnered	5 (21.7)
Divorced/separated/widowed	5 (21.7)
Education	
Less than high school	2 (8.7)
High school diploma/GED	13 (56.5)
Some college/technical	6 (26.1)
College degree or higher	2 (8.7)
Employment status	
Unemployed/on disability	20 (87.0)
Part-time	2 (8.7)
Full-time	1 (4.3)
Annual household income	
	20 (87.0)
$20,000–$39,999	3 (13.0)
≥ $40,000	0 (0.0)
Housing stability	
Stable housing	22 (95.7)
Unstable housing/homeless	1 (4.3)
Insurance status	
Public (Medicaid/Medicare)	23 (100)
Private	0 (0.0)
Uninsured	0 (0.0)
SMI Diagnosis	
Bipolar disorder	10 (43.5)
Schizophrenia	4 (17.4)
Schizoaffective disorder	6 (26.1)
Other SMI	3 (13.0)
CVD Condition(s) Reported*	
Hypertension	8 (34.8)
Diabetes	8 (34.8)
High cholesterol	2 (8.7)
Hypertension & Diabetes	2 (8.7)
Hypertension & Cholesterol	2 (8.7)
Diabetes/Hypertension/Cholesterol	1 (4.3)

*Participants reported more than one CVD condition.

Age presented as mean (SD). Percentages are based on valid responses. Demographics reflect a purposive sample of African American adults with co-occurring serious mental illness (SMI) and cardiovascular disease (CVD) recruited from the GPC/AAGPC study cohort.

Most participants reported annual household incomes below $20,000 and were unemployed or receiving disability benefits, reflecting limited economic resources. Nearly all relied on public insurance (Medicaid or Medicare), and a subset reported periods of housing instability or homelessness. Educational attainment varied, though few participants had completed college.

In terms of health status, bipolar I disorder emerged as the most common SMI diagnosis, followed by schizoaffective disorder and schizophrenia. Hypertension was the most frequently reported cardiovascular condition, often occurring alongside diabetes or other cardiometabolic conditions. These overlapping health challenges illustrate the cumulative disease burden within the sample.

Taken together, these characteristics highlight the profound structural and social disadvantage experienced by participants. Low income, unstable housing, and reliance on public benefits underscored the role of structural determinants in shaping both psychiatric and cardiovascular outcomes. These vulnerabilities provided important context for the syndemic processes that emerged in participants’ narratives.

Grounded theory analysis examines how African Americans with co-occurring serious mental illness (SMI) and cardiovascular disease (CVD) interpret and manage their health within the context of their social, cultural, and economic environments. Employing syndemic theory as a framework, this study highlights the layered impacts of these intersecting factors. The four primary themes—*Mental Illness as a Driver for Physical Illness, The underlying structural drivers of health behaviors, Structural and Environmental Influences on Health Behaviors and Distrust in the Healthcare System*—illuminate the complex interactions that shape participants’ lived experiences.

### Theme one: mental illness as a driver for physical illness

In line with grounded theory analysis, one central category that emerged was the reciprocal influence of psychiatric symptoms and cardiovascular illnes**s**. Participants articulated that mental illness not only undermined efforts to manage cardiovascular disease (CVD) but was also exacerbated by the physical decline associated with chronic conditions. This interdependency was described across the sample and illustrates the cyclical process of worsening health across both domains.

Depressive symptoms, hallucinations, and impaired concentration disrupted routines for diet, medication adherence, and exercise. One participant explained:

*“I eat a lot of salt … Yeah, that’s by choice. I know it’s bad for my blood pressure, but when I’m down I just don’t care.”*

Similarly, psychiatric symptoms created barriers to sustaining treatment plans:

*“I’ve been waking up every morning hearing voices and I have high cholesterol. Just living day by day.”*

A few participants discussed substance use as part of this cycle, framing it not as an isolated behavior but as a response to psychiatric distress that carried long-term cardiovascular consequences. A male participant with Schizophrenia explained: *“I had a family, I had a home … I just felt very depressed … and then the drugs came in.”* The onset of substance use worsened his hypertension and diabetes, further reinforcing the cycle of decline.

Conversely, participants also described physical illness as intensifying psychiatric distress. A man who had experienced a mini-stroke explained:

*“I suffered from a mini stroke … High blood pressure is one and stress is another.”*

Similarly, another said:

*“My depression makes it hard to take care of my heart … but when I can’t breathe or feel chest pain, my anxiety goes through the roof.”*

Another participant elaborated on this bidirectional link, noting that while physical health could be stabilized through medication, mental instability persisted and, in turn, disrupted cardiovascular self-management:

*“I can say that my mental piece is more challenging right now than my medical because I … even though I don’t like the medicine I still take the medicine for the medical piece which pretty much stabilizes my medical issues for now … But if my mental stability is not intact, it’s definitely going to affect my medical.”*

This underscores how mental illness served as both a driver and consequence of physical illness—reinforcing the core pattern of syndemic reciprocity.

Similarly, another participant contrasted the “mental” and “medical” aspects of their condition, describing how even consistent adherence to medical treatment could not offset the destabilizing effect of psychiatric symptoms:

*“I can say that my mental piece is more challenging right now than my medical … if my mental stability is not intact, it’s definitely going to affect my medical.”*

Together, these accounts illustrate how psychiatric instability directly undermines cardiovascular self-care, reinforcing a feedback loop of syndemic interdependency between mental and physical illness.

This dual challenge—navigating both mental and physical symptoms daily—illustrates the compounded burden of coexisting conditions, which syndemic theory suggests intensifies overall health challenges. Syndemic theory posits that co-occurring conditions heighten vulnerability to additional risks, and this analysis indicates how mental health challenges can foster behaviors that directly impact physical health.

Across interviews, participants emphasized reciprocity: *“My mental health is affecting my relationships and my physical health.”* These narratives illustrate how SMI and CVD function as mutually reinforcing conditions, creating cycles where psychiatric instability impairs health management, physical deterioration deepens distress, and each amplifies the other over time.

### Theme two: underlying structural drivers of health behaviors

Participants repeatedly emphasized how economic deprivation and structural inequities shaped their health decisions, often forcing survival needs to take priority over self-care. The daily struggle for housing, food, and financial stability was described as both a cause and a consequence of their health challenges, revealing how structural forces entangle mental and physical illness.

Most participants reported relying on Social Security benefits and food stamps, which they described as barely sufficient to cover basic expenses and inadequate to purchase nutritious food. As one participant explained: *“I have a fixed income … I eat whatever I can eat … just enough to get by.”* Another added: *“Vegetables are expensive … the good stuff costs you a lot more than the bad stuff.”* For many, the support they received allowed them to survive, but not to thrive. In their words, the benefits kept them afloat but did not enable them to manage their health in meaningful ways.

Housing emerged as another pressing concern. As one participant explained: *“Housing and money are a great stress for me, not my health.”* Homelessness was described as the “number one source of stress,” worsening depression and anxiety, which in turn contributed to high blood pressure and other CVD risks.

Food insecurity was a constant subtheme in these narratives. Rising prices and limited benefits pushed participants toward cheaper, calorie-dense foods:

*“I just cook when I eat eggs, I eat bread, stuff like that … all the food went up so I am not able to eat quality food.”*

While unhealthy diets worsened cardiovascular conditions, the stress of scarcity also exacerbated psychiatric symptoms, creating a dual burden. Explaining further, a female participant shared her daily struggle.

*“Food stamps help, but I still can’t afford fresh food … the stress makes my blood pressure worse and triggers my mood swings.”*

Unemployment further entrenched this cycle, often tied to both psychiatric instability and racial discrimination. One participant explained:

*“I don’t work … but when you Black you got a couple of strikes against you anyway. People see differently, and it plays a role on your health.”*

In this narrative, race and poverty intersect with serious mental illness to create systemic exclusion from stable work—leading to poverty, food insecurity, and poor health management.

These accounts demonstrate that structural deprivation is not background context but an active driver of the syndemic cycle. Social Security and food stamps provided just enough for participants to “get by,” but not to achieve health. Economic strain heightened psychiatric symptoms; psychiatric instability made sustained employment difficult; unemployment limited income and reduced access to quality food and housing; and these constraints worsened CVD risk. The reciprocal loop is clear: poverty worsens illness, illness entrenches poverty, and both reinforce each other in the context of systemic inequities. This theme underscores that for participants, the focus was on survival—not on health—a condition that leaves the syndemic cycle largely unbroken.

These experiences illustrate how economic deprivation, a core concept in syndemic theory, magnifies health vulnerabilities, particularly among individuals with co-occuring SMI and CVD. These structural scarcities were not experienced in the abstract; they were lived in place. Participants next situated scarcity within neighborhood conditions that made healthy choices practically unreachable.

### Theme three: structural and environmental influences on health

Participants described how the environments in which they lived—marked by stigma, neighborhood inequities, and limited access to nutritious food—directly influenced both mental and physical health. These structural conditions created chronic stress that worsened psychiatric symptoms while also driving cardiovascular risk.

Neighborhood stigma and surveillance were central to participants’ accounts. Being stopped, searched, or criminalized in their own communities generated both psychological distress and physiological consequences. A male participant explained:

*“Just by walking in the street … you can be stopped and frisked. They do things to keep the drugs out of their neighborhood and put the drugs in our neighborhood.”*

Another described the long shadow of an arrest:

*“When you go to apply for a job you got to put down that you was arrested … once they see you was arrested they don’t want to hire you. It’s a vicious cycle.”*

Participants linked these experiences to chronic stress, anxiety, and feelings of hopelessness, which in turn contributed to hypertension and cardiovascular deterioration. Living under constant suspicion and discrimination intensified their psychiatric symptoms while simultaneously placing strain on their cardiovascular systems.

Unequal access to food was another pervasive challenge. Participants contrasted food options in poorer Black neighborhoods with wealthier areas:

*“The stigma of being in a predominantly Black neighborhood … you’re being harassed every day by not only the police department, but by your local grocery stores. The grocery store in Brownsville is different from the grocery store in downtown Brooklyn.”*

Another explained: *“In the poor neighborhood, you got more sugar products than vegetables.”* Participants stressed that these limited options made it nearly impossible to follow the dietary advice they received for managing chronic conditions such as hypertension and diabetes. As one put it:

*“Why is it that the bad food costs less than the good food? That’s because they don’t care about people’s health … then the doctors can prescribe medicine. It’s all about the money.”*

The frustration of being unable to access healthy foods compounded psychiatric symptoms, while reliance on unhealthy diets worsened cardiovascular disease, creating a self-reinforcing cycle.

Housing stability occasionally offered protection, though it was precarious. One participant shared: *“I live in subsidized housing … that takes a lot of stress off.”* Yet, for others, the risk of losing housing was the greatest source of anxiety: *“Main objective [is to] keep a roof over my head, so I don’t be homeless again.”* For these individuals, fear of homelessness triggered psychiatric instability, while chronic stress and instability elevated blood pressure and compounded CVD risk.

These narratives demonstrate that environments are not neutral backdrops but active determinants of health, embedding stigma and scarcity into everyday life. Neighborhood-level criminalization increased psychiatric distress and triggered stress pathways that worsened cardiovascular outcomes. Food deserts and unequal access to nutritious options directly compromised participants’ ability to manage CVD, while reinforcing hopelessness and frustration that deepened mental health symptoms. Housing instability added another layer of stress, with homelessness described as the greatest driver of psychiatric decline and cardiovascular strain. Together, these accounts illustrate how structural and environmental contexts functioned as syndemic amplifiers, fueling the reciprocal interplay of SMI and CVD.

### Theme four: distrust in the healthcare system

A fourth theme centered on distrust of healthcare providers, especially within psychiatric care, which participants perceived as exploitative, profit-driven, and dismissive. This distrust was not incidental—it emerged as a patterned, cumulative response to repeated negative encounters. Grounded theory analysis revealed that such distrust had a reciprocal impact on both mental health and CVD management: avoidance of psychiatric care worsened psychiatric symptoms, which then undermined cardiovascular self-care; simultaneously, poor physical outcomes reinforced skepticism toward medical providers, deepening disengagement.

Participants frequently associated psychiatry with overmedication and commodification. One explained:

*“I don’t see the psychiatrist … because they’re always prescribing drugs.”*

Another participant with Schizophrenia questioned his position with his psychiatrist:

*“They just push pills on me … I don’t feel like they care about me as a person.”*

Another added:

*“I got a doctor that’s lying to me … he’s writing me prescriptions and I don’t know how this doctor is writing me a prescription.”*

These accounts illustrate how participants interpreted psychiatric care as inattentive to their lived realities, fueling avoidance and mistrust.

This skepticism also extended into cardiovascular care. Some participants described clinicians as inattentive or transactional, reinforcing feelings of exploitation. For example, one participant recounted how physicians emphasized prescriptions without discussing lifestyle or contextual barriers. The result was disengagement from both psychiatric and physical health services, leaving conditions to worsen in tandem.

Through constant comparison, the analytic pattern becomes clear: mistrust operates as a syndemic mechanism. Psychiatric avoidance leads to greater psychiatric instability, which erodes the capacity to adhere to CVD regimens. Conversely, poor cardiovascular outcomes confirm participants’ suspicion that providers are ineffective or uncaring, amplifying avoidance of care. This cyclical feedback produces what participants described as “day by day survival” rather than structured health management.

Participants repeatedly expressed the view that providers were motivated by profit rather than care. One participant explained:

*“Yes, yes, yes drugs … Before I had diabetes I don’t really think I had diabetes. But they give you diabetes drugs, then you got diabetes. They making money off the medication, off the diseases that they say I have that I don’t really believe.”*

This skepticism often led to disengagement. A female participant with bipolar 1 reflected:

*“I like to surface where we can sit down and talk … That to me is more beneficial than a pill. I haven’t met a psychiatrist yet that didn’t prescribe something.”*

Others described feeling exploited:

*“It’s all about the money amount.”*

However, a small number of participants also described moments of trust and empathy in their healthcare experiences. When providers took time to listen, explained treatment decisions, or demonstrated genuine concern, participants expressed appreciation and greater willingness to engage in care. One participant shared, *“My nurse, she really listens—she don’t just look at the chart, she asks how I’m doing.”* Another explained that having a consistent psychiatrist *“who talks to me like a person”* made it easier to stay on medication. These counter-narratives reveal that trust could be built when care felt relational rather than transactional. Yet even these positive encounters were framed as exceptions, dependent on individual provider relationships rather than systemic reliability. As a result, participants viewed trust as fragile—easily disrupted by bureaucracy, rushed visits, or perceived disrespect.

Mistrust extended into cardiovascular care as well, reinforcing cycles of disengagement and poor outcomes. Participants reported avoiding doctors or doubting prescriptions, which left both SMI and CVD unmanaged. This disengagement fueled further deterioration, deepening the conviction that the system was exploitative. In this way, healthcare distrust functioned as both a product of lived experiences and a driver of worsening health—amplifying the feedback loops that define syndemic interdependency.

### Grounded theory: conceptual model of syndemic interdependency

**Figure 1:**
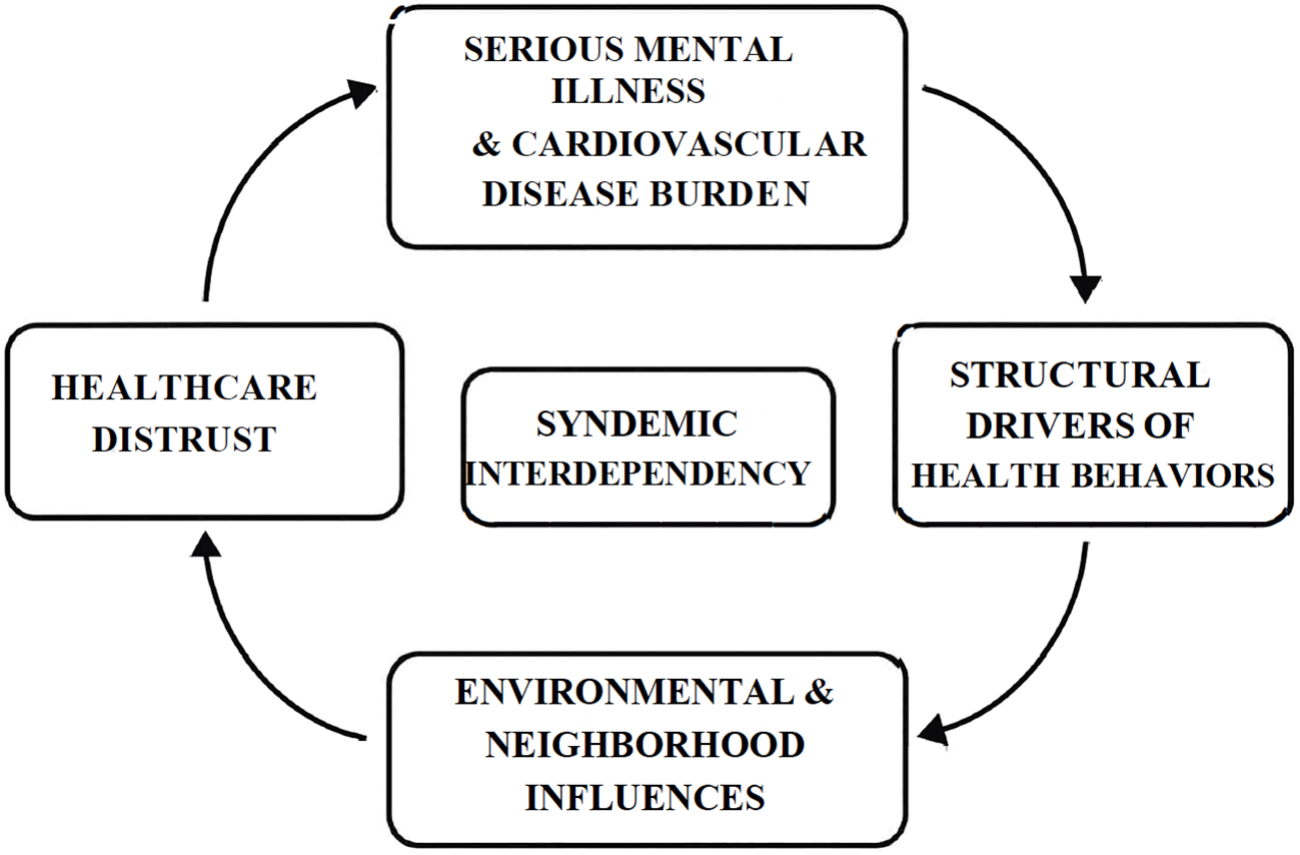
A graphic representing the grounded theory of Syndemic Interdependency. This diagram illustrates how the core concept, *Syndemic Interdependency*, is influenced by interconnected themes: Mental and Physical Health, Underlying structural drivers of health, Environmental Stressors and Healthcare Distrust,. Each theme is linked to the central concept, symbolizing the dynamic and compounding effects of these factors on participants’ health perceptions and behaviors.

Based on the four themes summarized in **Table 2**, and consistent with the expectations of grounded theory, this study generated a theoretical model of syndemic interdependency. This model explains how participants’ health perceptions and management strategies are shaped by the reciprocal interaction of mental and physical illness within a socio-structural framework. In this process, SMI and CVD are not parallel conditions but entangled in ways that mutually reinforce one another: psychiatric symptoms undermine cardiovascular self-management, while cardiovascular decline deepens psychiatric distress. Structural deprivation and environmental inequities amplify these conditions, and distrust in healthcare severs potential avenues for intervention. The outcome is a compounded health burden greater than the sum of its parts—an interdependent syndemic process rooted in structural marginalization (**Figure 1**).

**Table 2:** Emergent themes and representative quotes.

Theme	Description	Representative quote(s)
Theme 1: Mental Illness as Both Driver and Consequence of Physical Illness	Participants described reciprocal cycles where psychiatric symptoms disrupted CVD management, while CVD complications intensified psychiatric distress.	“My depression makes it hard to take care of my heart … but when I can’t breathe or feel chest pain, my anxiety goes through the roof.”
Theme 2: Structural Drivers of Health Behaviors	Reliance on public assistance, food insecurity, and economic strain amplified both mental and physical health challenges.	“Food stamps help, but I still can’t afford fresh food … the stress makes my blood pressure worse and triggers my mood swings.”
Theme 3: Environmental and Neighborhood Influences	Unstable housing, unsafe neighborhoods, and lack of healthy food outlets constrained participants’ ability to manage health.	“I stay in a shelter most nights—it’s not safe, and it’s impossible to eat healthy or rest.”
Theme 4: Distrust in the Healthcare System	Mistrust of providers and skepticism toward medications reinforced disengagement from both psychiatric and cardiovascular care.	“They just push pills on me … I don’t feel like they care about me as a person.”

This interdependency produces cyclical patterns of risk and coping, highlighting that addressing any single component in isolation is unlikely to improve outcomes. Instead, interventions must recognize the interconnections among psychiatric illness, cardiovascular disease, and the structural and relational forces that shape them. The analytic model underscores the expectation in grounded theory to move beyond description toward conceptual integration.

## Discussion

This grounded theory study, based on interviews with 23 African American adults living with co-occurring SMI and CVD, generated the theory of syndemic interdependency. Using syndemic theory as a sensitizing framework, we found that participants consistently described their health as shaped by reciprocal cycles of psychiatric and cardiovascular burden, amplified by structural disadvantage, environmental stressors, and distrust in healthcare. Syndemic interdependency captures how these forces operate together to bind individuals into self-perpetuating cycles of illness, as anticipated when grounded theory is applied with a syndemic lens (38, 39).

For example, the first theme revealed that participants viewed mental illness as both a driver and a consequence of physical illness. Psychiatric conditions such as schizophrenia, bipolar disorder, or schizoaffective disorder often undermined cardiovascular self-management, while the physical limitations of CVD intensified psychiatric distress. This reciprocity reflects syndemic theory’s proposition that health conditions interact synergistically within marginalized populations (38, 40). Participants described how unmanaged SMI led to poor diet, limited activity, or neglect of medical care, all of which heightened CVD risk, echoing Hanrahan et al. (41). The contribution of this study lies in demonstrating that participants themselves conceptualized comorbidities as intertwined, self-perpetuating conditions rather than separate illnesses. In doing so, the findings extend syndemic theory by illustrating the lived experience of reciprocity as a central feature of SMI–CVD comorbidity.

Building on this, the second theme highlighted how structural drivers of health behaviors intensified these health cycles. Participants frequently described reliance on Social Security, food stamps, and subsidized housing, which provided minimal stability but constrained their ability to access nutritious food or quality healthcare. This finding is consistent with studies linking poverty and food insecurity to syndemics of chronic illness (42, 43). Yet participants emphasized that while assistance programs were necessary, they seldom enabled health autonomy and often reinforced cycles of dependency. Psychiatric symptoms such as anxiety and depression were compounded by financial stress, amplifying the syndemic process. The contribution of this study is to show that economic marginalization does not merely serve as background context, but operates as an active amplifier of syndemic vulnerability, shaping health behaviors and constraining opportunities for recovery. This shifts the focus from individual adherence to the structural underpinnings of self-care.

The third theme demonstrated the profound influence of environmental stressors. Participants emphasized how unsafe housing, lack of healthy food outlets, and community violence acted as daily barriers to health, consistent with prior research linking neighborhood stressors to compounded psychiatric and cardiovascular risk (21, 44). Importantly, participants also described how stable housing reduced mental health stress and indirectly improved cardiovascular management. While Cohen et al. (19, 41) suggested that neighborhood interventions alone may be insufficient, these findings illustrate how even partial improvements in housing and safety can mitigate syndemic processes. The contribution here is to extend prior research by showing that environmental disadvantage functions not only as an external constraint but also as a determinant of how individuals prioritize survival over health, thereby reinforcing syndemic interdependency.

Finally, the fourth theme revealed the pervasive distrust in healthcare systems. Participants expressed skepticism toward psychotropic medications and perceived that providers prioritized pharmaceutical management over holistic care, reflecting broader patterns of medical mistrust (45, 46). In this study, however, mistrust itself functioned syndemically: disengagement from psychiatric care worsened mental illness, which undermined cardiovascular management, while declining cardiovascular health reinforced perceptions of neglect. Law et al. (47) argue that acknowledging distrust can open opportunities for improved relationships. The contribution of this study is to demonstrate how mistrust not only hinders engagement but becomes embedded in syndemic processes as a driver of disease synergy. This finding expands syndemic theory by highlighting mistrust as both a social determinant and a mechanism of syndemic amplification.

Although medication use was not the central focus of this analysis, nearly all participants reported being prescribed psychotropic and/or cardiovascular medications. Many described complex regimens, often involving antipsychotics, mood stabilizers, antihypertensives, and diabetes drugs. Thematic analysis revealed that medication experiences were central to participants’ perceptions of health and mistrust. Some described feeling overmedicated or questioned diagnostic accuracy, while others reported positive outcomes when medication was paired with relational trust and communication. These accounts align with prior work suggesting that perceived overmedication and side effects reinforce mistrust and nonadherence (48, 49). Future research should examine how treatment adherence and polypharmacy shape syndemic interdependency, particularly given the bidirectional physiological and psychosocial effects of psychotropic and cardiometabolic medications (50).

### Relative influence of mental and physical illness

Participants’ narratives also provided insight into the relative influence of mental versus physical illness in the syndemic cycle. While both were mutually reinforcing, psychiatric instability often served as the proximal driver of decline, disrupting motivation, self-care, and adherence. Cardiovascular symptoms, in turn, reinforced anxiety, depression, and stress, closing the feedback loop. In this sense, mental illness frequently functioned as the initiating destabilizing force, while physical illness acted as the sustaining amplifier—a pattern consistent with mutual causation rather than linear progression (42). This distinction underscores that SMI and CVD should be treated not as comorbid but as dynamically intertwined domains of the same syndemic process.

### Syndemic interdependency and theoretical contribution

Taken together, these findings demonstrate that comorbidity between SMI and CVD in African Americans is best understood not as parallel biomedical conditions but as a syndemic system. Syndemic interdependency, the theory generated here, advances understanding of how psychiatric and cardiovascular conditions, structural inequities, environmental disadvantage, and healthcare mistrust operate as interlocking forces that perpetuate poor health outcomes.

As anticipated in grounded theory, this study generated a theoretical model—syndemic interdependency—to explain how African Americans with co-occurring SMI and CVD experience and manage their health. This model demonstrates that these conditions are not parallel but reciprocally entangled: psychiatric symptoms disrupt cardiovascular self-management, while cardiovascular decline exacerbates psychiatric distress. Structural deprivation and environmental inequities magnify these cycles, and distrust in healthcare severs potential avenues for intervention. The result is a compounded health burden greater than the sum of its parts—an interdependent syndemic process rooted in structural marginalization.

The unique contribution of this study is to extend syndemic theory by showing how comorbidities are lived not simply as biomedical conditions but as mutually reinforcing cycles shaped by social and structural inequities. In doing so, the model highlights mistrust, polypharmacy, and environmental disadvantage as active drivers of syndemic amplification rather than background factors. Syndemic interdependency thus advances theory by integrating illness interaction, structural marginalization, and patient perspectives into a single explanatory framework.

### Extending prior models: from reciprocity to syndemic interdependency

While earlier research has documented reciprocal or bidirectional relationships between mental and physical illness—such as the mutual exacerbation of psychiatric distress and cardiovascular risk (2, 8)—our findings extend this understanding through the construct of *syndemic interdependency*. Rather than depicting SMI and CVD as two conditions influencing each other in isolation, Syndemic interdependency specifies how reciprocity is sustained and intensified by structural drivers (poverty, racism), environmental-based constraints, and institutional mistrust, turning co-occurrence into a durable system of dependency. It reframes reciprocity not as a dyadic exchange between diseases but as a web of mutually reinforcing biological, psychological, and structural forces that bind individuals into self-perpetuating cycles of illness. This conceptual extension advances syndemic theory (34, 40, 42) by elucidating how synergy operates *through* mechanisms of dependency and entanglement, thereby linking micro-level comorbidity processes with macro-level inequities in health and healthcare.

By reframing comorbidity through the lens of *syndemic interdependency*, this study highlights the need for interventions that move beyond single-disease management or symptom-based coordination. Because interdependency operates across structural, behavioral, and biological domains, effective responses must similarly act across levels—integrating medical care with mental-health support, community-based resources, and policy reforms that address the upstream social determinants sustaining the cycle. This approach underscores that reducing syndemic burden among individuals with co-occurring SMI and CVD requires not only clinical integration but also structural repair—policies that promote housing stability, economic inclusion, and trust in healthcare systems. Thus, *syndemic interdependency* provides both a conceptual and practical framework for multilevel intervention design and equity-focused health reform.

Although *syndemic interdependency* advances conceptual understanding of how mental and physical illnesses intertwine within structural inequities, further research is needed to refine and operationalize this construct. Quantitative studies could examine measurable indicators of interdependency—such as co-evolving trajectories of psychiatric and cardiovascular outcomes in the context of social adversity—while longitudinal and mixed-methods designs could illuminate causal pathways and temporal sequencing. Additionally, future inquiry should test how interventions that address multilevel determinants—ranging from care coordination to structural policy reform—can disrupt these interdependent cycles. By empirically extending this framework, researchers can translate *syndemic interdependency* from a grounded theoretical insight into a measurable construct with direct relevance for prevention, treatment, and health-equity implementation.

Taken together, these findings underscore the theoretical and practical value of *syndemic interdependency* while also acknowledging that this grounded model—derived from a specific sample and context—represents an interpretive framework requiring further testing and refinement.

### Implications for treatment and support

These findings suggest several directions for integrated intervention. First, clinical models should coordinate psychiatric and cardiovascular care through shared management plans, cross-disciplinary teams, and consistent patient–provider relationships to rebuild trust (2, 51). Second, addressing social determinants—such as food insecurity, housing instability, and exposure to community violence—is critical for sustained health improvement (52). Community-based interventions, peer support programs, and culturally grounded navigation services can bridge medical and psychosocial care gaps (53). Finally, policy reforms to strengthen insurance coverage for integrated behavioral and primary care and investments in neighborhood safety and housing stability are essential for breaking syndemic cycles (54). Collectively, these strategies align with syndemic theory by addressing the interacting medical, psychological, and structural dimensions of illness rather than treating them in isolation.

### Linking findings to existing intervention models

The framework of *syndemic interdependency* complements and extends current models of integrated care and multilevel intervention. For example, collaborative care models and behavioral health homes emphasize coordination across mental and physical health systems (53, 55), yet often under address the structural conditions that shape patient engagement and outcomes. Our findings suggest that interventions grounded in community-based participatory and structural competency approaches (56, 57) may more effectively disrupt the interdependent cycles described by participants. Embedding these principles within syndemic-informed frameworks can guide programs that simultaneously target clinical management, psychosocial support, and the social determinants that sustain inequity.

Beyond conceptual integration, this model offers practical utility: it provides a foundation for testing and designing interventions that address psychiatric and cardiovascular care simultaneously, while targeting upstream determinants such as food insecurity, neighborhood safety, and systemic racism. The following section outlines key limitations and considerations for future research.

### Limitations

This study offers valuable insights into the lived experiences of African American adults with co-occurring SMI and CVD; however, several limitations should be acknowledged. First, the relatively small sample size (N = 23) and recruitment from a single urban setting may limit the transferability of findings to other geographic or demographic contexts. Second, while SMI diagnoses were verified through structured clinical interviews within the GPC registry, CVD status was self-reported. Although self-report data are widely used and considered reliable for chronic conditions such as hypertension and diabetes, this asymmetry introduces potential reporting bias and may be subject to recall. Similarly, selection bias from recontacting prior cohort participants and Zoom-based interviewing possibly excluding those without stable access. Third, because the sampling strategy purposively included only participants living with both SMI and CVD, individuals with SMI alone were excluded. Although this limits direct comparison across diagnostic groups, the approach was intentional to illuminate the interdependencies that emerge when psychiatric and cardiovascular conditions coexist. Fourth, the study did not include quantitative assessments of social determinants such as income, education, or housing instability within the broader population, which could have provided additional contextual grounding. Fifth, medication regimens were not systematically abstracted from participants’ clinical records; instead, perceptions and self-reported experiences with medications were the analytic focus. This approach aligns with the qualitative design, emphasizing lived experience over biomedical chart abstraction. Finally, the sample, though demographically diverse, reflected a subset of younger, socioeconomically disadvantaged African Americans with co-occurring SMI and CVD. As a purposive, theory-generating sample, these findings may not be generalizable to the broader population but instead illuminate mechanisms of syndemic interdependency among those most exposed to structural vulnerability.

Despite these limitations, the depth and richness of participants’ narratives lend strong credibility and analytic insight into how co-occurring psychiatric and cardiovascular illness are shaped by structural disadvantage, environmental stressors, and medical mistrust. The use of grounded theory methodology enhances the study’s trustworthiness by allowing the emergent concept of *syndemic interdependency* to be derived directly from participants’ lived experiences, offering a theoretically grounded and empirically meaningful framework for future inquiry.

Taken together, these findings underscore the value of centering the lived experiences of marginalized populations to reveal how structural inequities, psychological burden, and chronic disease intersect. By introducing syndemic interdependency as a framework, this study advances understanding of comorbidity beyond individual risk factors to encompass the systemic conditions that sustain health disparities. Building on this foundation, future research should also consider how these syndemic processes manifest across different racial and ethnic groups. Although this study focused exclusively on African American adults, the decision to center this population was intentional, given the disproportionate burden of serious mental illness and cardiovascular disease within this community, compounded by structural inequities such as racism, economic hardship, and limited access to care. However, comparative syndemic studies remain scarce, and little is known about whether the interaction between psychiatric and physical health conditions, trust and environmental determinants differs across populations. Future research that examines these relationships in racially and ethnically diverse samples will help identify shared versus context-specific mechanisms and inform more targeted models of prevention and care.

## Conclusion

This grounded theory study contributes to the growing body of syndemic research by articulating *syndemic interdependency* as a mechanism linking co-occurring psychiatric and cardiovascular illness within structurally marginalized contexts. Participants’ narratives revealed that mental and physical health are not merely reciprocally linked but are interwoven through complex, self-reinforcing systems of social, psychological, and biological constraint. This framework underscores that health inequities among individuals with SMI and CVD cannot be addressed through clinical treatment alone; they require coordinated, multilevel strategies that simultaneously target behavioral health, medical care, and the social determinants that perpetuate illness cycles. By integrating patient perspectives with syndemic theory, this study offers a conceptual foundation for interventions that seek not only to manage disease but to dismantle the structural dependencies that sustain it.

## Data Availability

The raw data supporting the conclusion of this article is not publicly available to protect participant confidentiality and privacy. Requests to access the datasets should be directed to the corresponding author.
